# Testing persuasive messages about booster doses of COVID-19 vaccines on intention to vaccinate in Australian adults: A randomised controlled trial

**DOI:** 10.1371/journal.pone.0286799

**Published:** 2023-06-02

**Authors:** Maryke S. Steffens, Bianca Bullivant, Jessica Kaufman, Catherine King, Margie Danchin, Monsurul Hoq, Mathew D. Marques

**Affiliations:** 1 National Centre for Immunisation Research and Surveillance, Kids Research, Sydney Children’s Hospitals Network, Westmead, NSW, Australia; 2 Faculty of Medicine and Health, The Children’s Hospital at Westmead Clinical School, The University of Sydney, Sydney, NSW, Australia; 3 Vaccine Uptake Research Group, Murdoch Children’s Research Institute, Melbourne, VIC, Australia; 4 Department of Paediatrics, University of Melbourne, Melbourne, VIC, Australia; 5 Department of General Medicine, Royal Children’s Hospital, Melbourne, VIC, Australia; 6 Clinical Epidemiology and Biostatistics Unit, Murdoch Children’s Research Institute, Parkville, Australia; 7 The National Child Health Poll, The Royal Children’s Hospital, Parkville, Australia; 8 School of Psychology and Public Health, La Trobe University, Melbourne, VIC, Australia; Brigham and Women’s Hospital, UNITED STATES

## Abstract

**Introduction:**

Achieving high COVID-19 vaccine booster coverage is an ongoing global challenge. Health authorities need evidence about effective communication interventions to improve acceptance and uptake. This study aimed to test effects of persuasive messages about COVID-19 vaccine booster doses on intention to vaccinate amongst eligible adults in Australia.

**Methods:**

In this online randomised controlled trial, adult participants received one of four intervention messages or a control message. The control message provided information about booster dose eligibility. Intervention messages added to the control message, each using a different persuasive strategy, including: emphasising personal health benefits of booster doses, community health benefits, non-health benefits, and personal agency in choosing vaccination. After the intervention, participants answered items about COVID-19 booster vaccine intention and beliefs. Intervention groups were compared to the control using tests of two proportions; differences of ≥5 percentage points were deemed clinically significant. A sub-group analysis was conducted among hesitant participants.

**Results:**

Of the 487 consenting and randomised participants, 442 (90.8%) completed the experiment and were included in the analysis. Participants viewing messages emphasising non-health benefits had the highest intention compared to those who viewed the control message (percentage point diff: 9.0, 95% CI -0.8, 18.8, *p* = 0.071). Intention was even higher among hesitant individuals in this intervention group compared to the control group (percentage point diff: 15.6, 95% CI -6.0, 37.3, *p* = 0.150). Conversely, intention was lower among hesitant individuals who viewed messages emphasising personal agency compared to the control group (percentage point diff: -10.8, 95% CI -33.0, 11.4, *p* = 0.330), although evidence in support of these findings is weak.

**Conclusion:**

Health authorities should highlight non-health benefits to encourage COVID-19 vaccine booster uptake but use messages emphasising personal agency with caution. These findings can inform communication message development and strategies to improve COVID-19 vaccine booster uptake.

**Clinical trial registration:** Registered with the Australian New Zealand Clinical Trials Registry (ACTRN12622001404718); trial webpage: https://www.anzctr.org.au/ACTRN12622001404718.aspx

## Introduction

COVID-19 vaccination has been critical for controlling the COVID-19 pandemic by protecting vulnerable individuals from severe disease, safeguarding health systems and helping return society to normal functioning [1, 2]. Booster doses of COVID-19 vaccines (additional doses given after completion of the two-dose primary course) are necessary to provide ongoing immunity to SARS-CoV-2 and offer increased protection against severe disease [3].

Suboptimal uptake of COVID-19 booster doses has occurred globally. In Australia, completion of a primary course of COVID-19 vaccine for individuals ≥16 years is >96%, but booster dose (third dose) coverage has stalled at just over 70% [4]. In other countries, the United Kingdom (UK) has achieved approximately 70% booster dose coverage in the eligible population [5], while Europe and the United States (US) have achieved just over 50% and 43% respectively [6, 7].

While many factors can contribute to low vaccine uptake, including both access and acceptance barriers, low motivation may continue to act as a barrier to uptake if not addressed [8]. This has been the experience with other pandemic vaccines, for example the 2009 H1N1 influenza pandemic vaccine [9, 10]. There are a range of factors that influence motivation to receive a COVID-19 vaccine. Both personal and collective health benefits have been found to shape acceptance and motivation [11–13], building on evidence from strategies to increase vaccine uptake more broadly [14–16]. Non-health benefits, i.e. the broader benefits of vaccination beyond direct protection from disease such as ability to travel, have been found to be motivating factors [12, 17]. Attaching importance to certain moral values, such as individual liberties and the agency or freedom to make personal health decisions, have been linked to hesitancy about COVID-19 vaccines [18] as well as routine vaccines [19].

Considering these findings, emphasising motivational factors in persuasive messaging could potentially be a successful strategy to support higher intention to receive a COVID-19 vaccine. There is evidence that persuasive messages can support higher intentions towards vaccination in general [8, 20, 21]. For COVID-19 vaccination specifically, a systematic review has found that messages emphasising personal or community health benefits of vaccination can be effective in supporting higher intentions, although results are mixed [22]. Three survey experiments in US and UK adults comparing the effect of a range of messages on intention to receive a COVID-19 vaccine found that messages emphasising personal health benefits of vaccination have a larger effect than those emphasising benefits to others [13, 23, 24]. By contrast, another large survey experiment in US adults found that messages emphasising non-health benefits (such as freedom from public health restrictions) and community benefits were effective. The community messages were the most effective of all, while those emphasising personal health benefits were not more effective than the control [25]. Evidence of the effectiveness of messaging promoting altruistic behaviour (i.e. behaviour beneficial to the community) on COVID-19 vaccine intention has also been found in other experiments in US and UK adults [26, 27]. There is limited research investigating the effectiveness of messages emphasising personal agency in making COVID-19 vaccination decisions.

Given the current state of evidence, it remains unclear what types of messages have the greatest effect on intention to receive a booster dose of COVID-19 vaccine. This is especially the case in populations that have experienced relatively low risk of encountering COVID-19, combined with restrictive public health measures. These were the conditions experienced in Australia at the time of this experiment, where COVID-19 case numbers were relatively low during the first two years of the pandemic, while public health restrictions, such as border closures and lockdowns, were applied liberally due to the country’s pursuit of a COVID-19 elimination strategy [28]. Such evidence can inform communications from health authorities and other stakeholders to encourage uptake of COVID-19 booster doses, especially written communications (e.g. emails), which were used to communicate with the public in Australia during the COVID-19 vaccine rollout. This study aimed to compare the effect of persuasive messages on intention to get a booster dose of COVID-19 vaccine in Australian adults. This study is part of a larger study investigating factors influencing COVID-19 vaccine acceptance in Australia and elsewhere [11, 12, 29, 30] and developing messaging to support acceptance of COVID-19 vaccines in various populations [31].

## Methods

### Experimental design

This was a parallel group, randomised, controlled, online experiment comparing intention to receive a COVID-19 vaccine booster dose and beliefs about COVID-19 vaccine booster doses between subjects after receiving persuasive message interventions. This study obtained ethics approval from the Sydney Children’s Hospitals Network Human Research Ethics Committee (2021/ETH00181). The study aims, methods, and data analysis plan were pre-registered on the Open Science Framework (https://osf.io/xajmz). In response to the skewed distribution of responses, a post hoc variation to the pre-registration was made to analyse intention to vaccinate and participant responses to belief items as dichotomous variables. See S1 File for original frequency distribution of intention to vaccinate. The trial was retrospectively registered with the Australian New Zealand Clinical Trials Registry (ACTRN12622001404718, https://www.anzctr.org.au/ACTRN12622001404718.aspx).

### Participants

Participants were adults 18 years or older residing in Australia who had received at least one primary dose of a COVID-19 vaccine but had not yet received a booster (third) dose and had access to the internet. Research company Quality Online Research (QOR) recruited a random sample of participants via email invitation from its accredited online panel. The QOR panel has >85,000 active members; original panel members were recruited via the Australia Post Lifestyle Survey, distributed to all Australian households. Ongoing recruitment is by invitation only. The panel reflects Australian Bureau of Statistics census data by age, gender, and state. Participants took part in the study via an online portal hosted by the research company and were offered points as incentive for participation equivalent to between AUD$1.00-$2.00, redeemable as payouts by PayPal, eGift cards or cheques. Participants gave written (digital) informed consent. Participants were recruited between 17–24 December 2021, with recruitment stopping when targets were met.

For context, at the time of data collection, the Australian population had experienced multiple rounds of restrictions on movement, limits on indoor and outdoor gatherings, closure of restaurants, gyms and non-essential retail, and interstate and international border closures. Approximately two months prior to data collection (in October 2021), people living in the states of New South Wales and Victoria had exited strict and lengthy lockdowns in response to the Delta wave. In December 2021, domestic border closures had started to lift, however strict international border closures, put in place in March 2020 to prevent people from leaving and entering the country, were still in place at the time. In December 2021, Australia was anticipating the occurrence of a further Omicron wave, with cases rising rapidly [32, 33].

### Pre-intervention survey items

After consenting, participants provided demographic information (age, gender, education, and state of residence). They were asked to respond to screening items about their COVID-19 vaccination status (‘Have you received a first/second/third dose of a COVID-19 vaccine?’, response options = yes/no). Participants were asked to indicate their hesitancy towards COVID-19 vaccines with a single item (‘How much do you agree with the following statement: “I feel hesitant about COVID-19 vaccines”‘, response options were on a 5-point scale from strongly disagree to strongly agree) informed by previous vaccination research [34, 35]. This item was used to categorise the participants into vaccine hesitant and accepting participants. Responses were recoded where (strongly agree, slightly agree, neither agree nor disagree) = hesitant, and (slightly disagree, strongly disagree) = accepting.

### Intervention

Participants received one of four intervention messages according to their randomised group. The intervention was a short piece of written material (a message of approximately 70–140 words) designed to encourage uptake of booster doses of COVID-19 vaccines. The material was modified from a public email communication campaign disseminated by an Australian health authority in November 2021. These modifications were made by the research team, based on the current state of evidence on the types of messages that may have an effect on intention to vaccinate. The control message informed recipients of eligibility requirements for a booster dose of COVID-19 vaccine. The four intervention messages added to this message, each using a different persuasive strategy. Table 1 shows the full intervention messages. The *’Personal health benefits’* message emphasised the reduction in risk of becoming infected, sick or dying that getting a booster dose of COVID-19 vaccine offers. The *’Community health benefits’* message emphasised the altruistic nature of receiving a COVID-19 vaccine, i.e., the reduction in risk of giving the virus to family members or people in the community. The *’Non-health benefits’* message emphasised the broader benefits that getting vaccinated could bring such as the possibility of freedom from future public health restrictions. The *’Personal agency’* message emphasised the control over one’s health gained by choosing vaccination. Survey software required participants to view the intervention text for a minimum of 30 seconds.

**Table 1:** Intervention messages.

Message name	Full text
***Control***	**Get your COVID-19 vaccine booster shot** If your second dose of a COVID-19 vaccine was more than 5 months ago, you can now receive a booster vaccination. You can check when you received your second dose by looking at your immunisation history statement or COVID-19 vaccination certificate.
***Personal health benefits***	**Protect your health, get your COVID-19 vaccine booster shot** If your second dose of a COVID-19 vaccine was more than 5 months ago, you can now receive a booster vaccination. Your protection from COVID-19 after vaccination reduces over time. Getting a booster vaccination will reduce the risk that you get infected, become very sick, or die from COVID-19. Booster doses of COVID-19 vaccine give you protection, no matter how old you are. You can check when you received your second dose by looking at your immunisation history statement or COVID-19 vaccination certificate. Remember, getting a booster dose of COVID-19 vaccine is the best way to keep protecting yourself.
***Community health benefits***	**Protect people you care about, get your COVID-19 vaccine booster shot** If your second dose of a COVID-19 vaccine was more than 5 months ago, you can now receive a booster vaccination. Protection from COVID-19 after vaccination reduces over time. No matter how old you are, getting a booster vaccination not only protects you, it also reduces your risk of giving the virus to your family members or people in the community, who could get sick and die from COVID-19. You can check when you received your second dose by looking at your immunisation history statement or COVID-19 vaccination certificate. Remember, getting a booster dose of COVID-19 vaccine is the best way to keep protecting all of us.
***Non-health benefits***	**Help get life back to normal. Get your COVID-19 vaccine booster shot** If your second dose of a COVID-19 vaccine was more than 5 months ago, you can now receive a booster vaccination. COVID-19 has stopped us from living our lives as freely as we used to. We’ve been locked down, and not been able to travel, go to weddings or funerals, and see family and friends. When you get a booster dose of COVID-19 vaccine, you’re helping to reduce the chance that restrictions return. You can check when you received your second dose by looking at your immunisation history statement or COVID-19 vaccination certificate. Remember, while you can’t do it alone, getting a booster vaccination is the best way for you to help make sure we can all keep living freely.
***Personal agency***	**Take control of your health. Get your COVID-19 vaccine booster shot** If your second dose of a COVID-19 vaccine was more than 5 months ago, you can now receive a booster vaccination. Getting a booster vaccination is not mandatory. It’s a personal choice–one that gives you control of your health and lets you protect the people you care about. We all want the freedom to make our own decisions about our health. When you get a booster dose of COVID-19 vaccine, you’re taking charge and choosing the best option to protect yourself, your family, and your community. You can check when you received your second dose by looking at your immunisation history statement or COVID-19 vaccination certificate. Remember, it’s in your hands to make the right decision for yourself and the people you care about.

### Post-intervention outcome measures

Immediately after the intervention, participants responded to items measuring outcomes. The primary outcome measure was intention to receive a COVID-19 vaccine booster dose. This was assessed with a single item (‘How likely is it that you will get a booster dose of COVID-19 vaccine?’, with 5 response options: definitely, probably, I’m not sure, probably not, definitely not), consistent with survey questions used in previous research [36]. Responses were transformed into a dichotomous variable where (definitely, probably) = ’intends to receive a COVID-19 vaccine’ and (I’m not sure, probably not, definitely not) = ’does not intend to receive a COVID-19 vaccine’. Participants then answered an attention check question (‘Please select strongly disagree for this item’, response options ranged from strongly disagree to strongly agree).

Secondary outcome measures were beliefs about COVID-19 vaccine booster doses. These were assessed by asking participants to indicate their agreement with belief statements about COVID-19 vaccine booster dose safety, effectiveness, necessity for protecting one’s own health, necessity for protecting others’ health, and risks associated with not vaccinating (response options were a 5-point scale: strongly disagree, slightly disagree, neither agree nor disagree, slightly agree, strongly agree. See S1 File for belief statements). Responses were transformed into dichotomous variables where (Strongly disagree, Slightly disagree, Neither agree nor disagree) = ’does not agree with belief’ and (Strongly agree, Slightly agree) = ’agrees with belief’.

### Sample size

The study aimed to recruit 480 participants to ensure a sample size of 430 participants, allowing for a drop-out/poor quality response rate of approximately 10%. The sample size was calculated using G*Power (an a priori power analysis tool) to estimate an effect size of Cohen’s d = 0.2 (the difference between two independent means) [37] with a specified power of 80% at 0.01 level of significance using analysis of variance (ANOVA) tests to compare outcomes between intervention groups, as specified in the pre-registered protocol. In line with the post hoc variation to analyse participant’s intention to vaccinate and belief items as dichotomous variables, the sample size of 85 participants per intervention group was adequate to estimate a difference in proportion of 15 percentage points or more between two groups using two-sample test of proportion (z-test) with a level of significance of 0.05 and power of 80%. Participants with incomplete surveys or who failed to answer the quality control attention check question correctly were excluded.

### Randomisation

**Fig 1:**
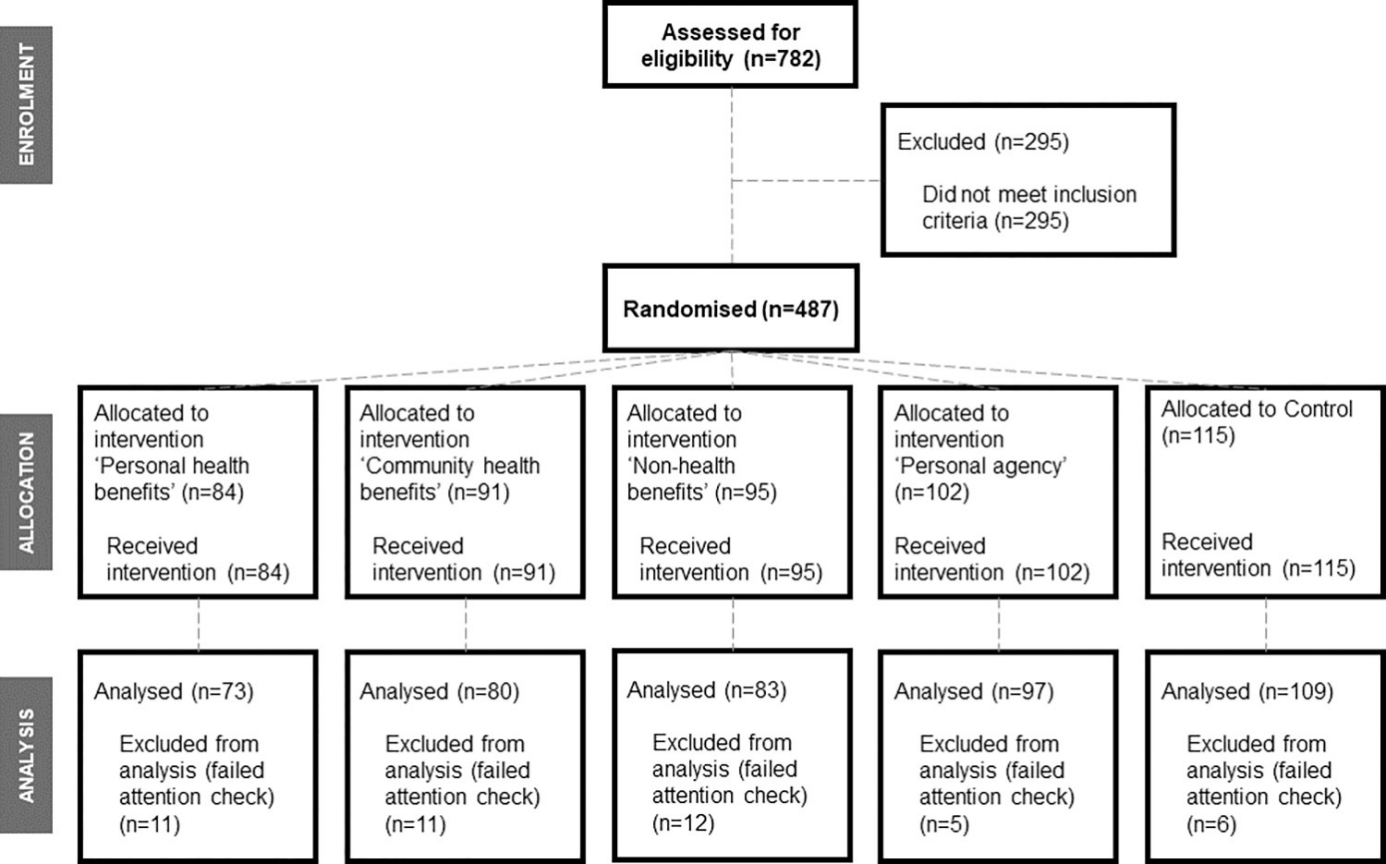
Flow diagram showing progress of participants through the online experiment.

Participants were randomly assigned at recruitment via a randomisation sequence embedded within the online system to receive one of the four intervention messages or the control message. Because some participants were excluded after randomisation (they either failed the attention check or did not complete the experiment, see Fig 1), a second randomisation sequence was generated to ensure intervention and control groups were of equal size. This sequence prioritised the least filled group when 80% of the recruitment was complete.

### Statistical methods

The difference in proportion of participants intending to receive a COVID-19 vaccine and agreeing with COVID-19 vaccine booster dose belief statements was compared between the control group and each of the four intervention groups using tests of two proportions (z test). A subgroup analysis included data from vaccine hesitant participants only.

Previous studies have shown that an effect size of ≥5 percentage points may have practical (clinical) significance [38–40]. Hence, when reporting differences in intention and beliefs for each intervention group compared to the control group, differences of ≥5 percentage points were considered practically meaningful from a public health perspective, regardless of the p value [41]. The difference in proportion was interpreted with respect to the upper and lower limit of the confidence intervals.

All analyses were conducted using SPSS (Statistical Package for Social Sciences, version 27).

### Role of the funding source

This research was funded by a grant from NSW Health. The sponsor had no role in the study design; in the collection, analysis and interpretation of data; in the writing of the report; or in the decision to submit the article for publication.

## Results

Of the 487 participants who consented and were randomised, 442 (90.8%) completed the experiment, answered the attention check question correctly, and were included in the analysis. Fig 1 shows the progress of participants through the online experiment.

Participants had a mean age of 51.14 years (*SD* 16.32); 51.1% (226/442) were female; 97.5% (431/442) had reported having received a second dose of COVID-19 vaccine; 37.8% (167/442) reported feeling hesitant about COVID-19 vaccines. Table 2 shows participant characteristics between groups, which are similar in age, gender, education, state, completion of second dose, or hesitancy between groups.

**Table 2:** Participant characteristics at baseline.

Group	All	Control	Personal health benefits	Community health benefits	Non-health benefits	Personal agency
**Total n**	442	109	73	80	83	97
**How old are you? n (%)**						
18–24	21 (4.8)	7 (6.4)	4 (5.5)	5 (6.3)	2 (2.4)	3 (3.1)
25–29	22 (5.0)	7 (6.4)	1 (1.4)	5 (6.3)	1 (1.2)	8 (8.2)
30–39	81 (18.3)	25 (22.9)	16 (21.9)	15 (18.8)	15 (18.1)	10 (10.3)
40–49	83 (18.8)	20 (18.3)	15 (20.5)	13 (16.3)	18 (21.7)	17 (17.5)
50–59	86 (19.5)	17 (15.6)	17 (23.3)	16 (20.0)	15 (18.1)	21 (21.6)
60–69	76 (17.2)	17 (15.6)	11 (15.1)	10 (12.5)	15 (18.1)	23 (23.7)
70+	73 (16.5)	16 (14.7)	9 (12.3)	16 (20.0)	17 (20.5)	15 (15.5)
**What is your gender? n (%)**						
Female	226 (51.1)	53 (48.6)	33 (45.2)	40 (50.0)	46 (55.4)	54 (55.7)
Male	213 (48.2)	55 (50.5)	39 (53.4)	40 (50.0)	37 (44.6)	42 (43.3)
Not specified	3 (0.7)	1 (0.9)	1 (1.4)	0 (0.0)	0 (0.0)	1 (1.0)
**What is the highest level of education or training you have completed? n (%)**						
Did not attend school	2 (0.5)	0 (0.0)	0 (0.0)	0 (0.0)	1 (1.2)	1 (1.0)
Year 12 or below	145 (32.8)	33 (30.3)	29 (39.7)	26 (32.5)	23 (27.7)	34 (35.1)
University degree	175 (39.6)	49 (45.0)	23 (31.5)	30 (37.5)	35 (42.2)	38 (39.2)
Other non-school qualifications	115 (26.0)	25 (22.9)	21 (28.8)	24 (30.0)	22 (26.5)	23 (23.7)
Prefer not to answer	5 (1.1)	2 (1.8)	0 (0.0)	0 (0.0)	2 (2.4)	1 (1.0)
**Where do you currently live (Australian state or territory)? n (%)**						
NSW	139 (31.4)	37 (33.9)	16 (21.9)	21 (26.3)	30 (36.1)	35 (36.1)
QLD	82 (18.6)	17 (15.6)	16 (21.9)	20 (25.0)	10 (12.0)	19 (19.6)
VIC	123 (27.8)	31 (28.4)	22 (30.1)	26 (32.5)	23 (27.7)	21 (21.6)
ACT	2 (0.5)	1 (0.9)	0 (0.0)	0 (0.0)	0 (0.0)	1 (1.0)
SA	41 (9.3)	5 (4.6)	9 (12.3)	7 (8.8)	7 (8.4)	13 (13.4)
WA	39 (8.8)	14 (12.8)	8 (11.0)	3 (3.8)	9 (10.8)	5	(5.2)
TAS	16 (3.6)	4 (3.7)	2 (2.7)	3 (3.8)	4 (4.8)	3 (3.1)
NT	0	0	0	0	0	0
**Have you received a second dose of a COVID-19 vaccine? n (%)**						
Yes	431 (97.5)	105 (96.3)	72 (98.6)	77 (96.3)	83 (100.0)	94 (96.9)
No	11 (2.5)	4 (3.7)	1 (1.4)	3 (3.8)	0 (0.0)	3 (3.1)
**How much do you agree with the following statement: “I feel hesitant about COVID-19 vaccines.” n (%)**						
Disagree	275 (62.2)	71 (65.1)	49 (67.1)	50 (62.5)	50 (60.2)	55 (56.7)
Agree	167 (37.8)	38 (34.9)	24 (32.9)	30 (37.5)	33 (39.8)	42 (43.3)

### Primary outcome (Intention to vaccinate)

**Fig 2:**
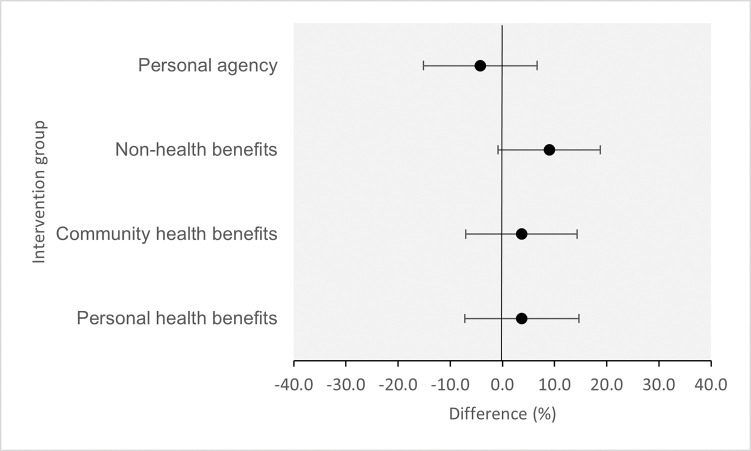
Difference in intention to vaccinate in each intervention group compared to the control group.

Most participants (84.6%, 374/442) indicated a positive intention to get a booster dose of COVID-19 vaccine. Table 3 shows intention to vaccinate, compared between groups. All intervention groups had a qualitatively higher intention to vaccinate compared to the control group except one (see Fig 2). Participants who viewed the message emphasising non-health benefits showed the largest difference compared to the control group (percentage point diff: 9.0, 95% CI -0.8, 18.8, *p* = 0.071), although the evidence in support of this finding is weak. Participants who viewed the message emphasising personal agency had a slightly lower intention compared to the control group (percentage point diff: -4.2, 95% CI -15.1, 6.7, *p* = 0.445), although there is a lack of evidence in support of this finding.

**Table 3:** Comparing intention to vaccinate between intervention groups and the control group.

Group	Total n	Definitely/probably intend on getting a booster dose	%	Diff^§^	95% CI Lower	95% CI Upper	*p*
Control	109	90	82.6	-	-	-	-
Personal health benefits	73	63	86.3	3.7	-7.2	14.7	0.500
Community health benefits	80	69	86.2	3.7	-7.0	14.3	0.494
Non-health benefits	83	76	91.6	9.0*	-0.8	18.8	0.071
Personal agency	97	76	78.4	-4.2	-15.1	6.7	0.455
All participants	442	374	84.6				

^**§**^ Percentage point difference

*Indicates a difference of ≥5 percentage points between the intervention group and the control group

**Fig 3:**
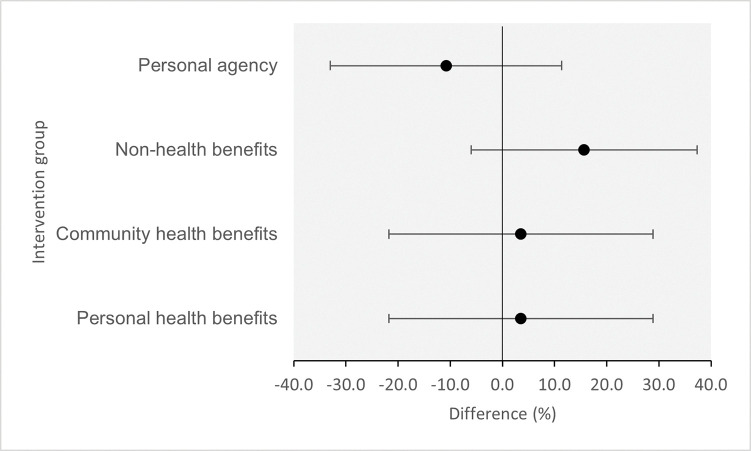
Difference in intention to vaccinate in each intervention group compared to the control group in hesitant participants (sub-analysis).

In the sub-analysis of hesitant participants, more than half of participants (64.7%; 108/167) intended to get a booster dose of COVID-19 vaccine; this proportion is lower than for the whole study population. Table 4 shows intention to vaccinate in hesitant participants, compared between groups. As with the whole population, all intervention groups had a qualitatively higher intention to vaccinate compared to the control group except one (see Fig 3). Participants who viewed the message emphasising non-health benefits showed a higher intention compared to the control group (percentage point diff: 15.6, 95% CI -6.0, 37.3, *p* = 0.150), while participants who viewed the personal agency message had a lower intention compared to the control group (percentage point diff: -10.8, 95% CI -33.0, 11.4, *p* = 0.330), although there is a lack of evidence in support of these findings. Results for non-hesitant participants are reported in S1 File.

**Table 4:** Comparing intention to vaccinate between intervention groups and the control group in hesitant participants (sub-analysis).

Group	Total n	Definitely/probably intend on getting a booster dose	%	Diff^§^	95% CI Lower	95% CI Upper	*p*
Control	38	24	63.2	-	-	-	-
Personal health benefits	24	16	66.7	3.5	-21.8	28.9	0.779
Community health benefits	30	20	66.7	3.5	-21.8	28.9	0.764
Non-health benefits	33	26	78.8	15.6*	-6.0	37.3	0.150
Personal agency	42	22	52.4	-10.8*	-33.0	11.4	0.330
All participants	167	108	64.7				

^**§**^ Percentage point difference

*Indicates a difference ≥5% between the intervention group and the control group

### Secondary outcome (Beliefs)

Most participants agreed that booster doses are safe (71.7%, 317/442), prevent disease (75.3%, 333/442), protect their health (78.7%, 348/442), protect the health of others (77.4%, 342/442). A slightly smaller majority agreed that they were at risk of getting COVID-19 without a booster dose (63.8%, 282/442).

Table 5 shows COVID-19 vaccine booster dose beliefs, compared between groups. Three of the intervention groups showed differences ≥5 percentage points in their agreement with some beliefs compared to the control, although there is a lack of evidence in support of these findings. Participants who viewed the message emphasising personal health benefits showed a higher agreement compared to the control group with beliefs about COVID-19 booster dose safety (percentage point diff: 5.1, 95% CI -7.6, 17.9, *p* = 0.424) and the necessity of booster doses for protecting other people’s health (percentage point diff: 7.0, 95% CI -5.4, 19.3, *p* = 0.266). Participants who viewed the message emphasising non-health benefits showed a higher agreement compared to the control with beliefs about booster doses being effective (percentage point diff: 7.6, 95% CI -4.4, 19.6, *p* = 0.210) and the necessity of booster doses for protecting other people’s health (percentage point diff: 6.7, 95% CI -5.2, 18.6, *p* = 0.266). Participants who viewed the message emphasising personal agency showed lower agreement compared to the control with beliefs about COVID-19 booster dose safety (percentage point diff: -5.2, 95% CI -17.7, 7.2, *p* = 0.404).

**Table 5:** Comparing beliefs between intervention groups and the control group.

Belief	Strongly/slightly agree		Diff^§^	95% lower	95% upper	*p*
	n	(%)				
**“Booster doses of COVID-19 vaccine are safe”**						
Control (n = 109)	81	(74.3%)	ref			
Personal health benefits (n = 73)	58	(79.5%)	5.1*	-7.6	17.9	0.424
Community health benefits (n = 80)	80	(75.0%)	0.7	-12.0	13.4	0.914
Non-health benefits (n = 83)	61	(73.5%)	-0.8	-13.5	11.9	0.898
Personal agency (n = 97)	37	(69.1%)	-5.2*	-17.7	7.2	0.404
All (n = 442)	317	(71.7%)				
**“Booster doses of COVID-19 vaccine do a good job preventing disease”**						
Control (n = 109)	81	(74.3%)	ref			
Personal health benefits (n = 73)	55	(75.3%)	1.0	-12.0	14.1	0.875
Community health benefits (n = 80)	58	(72.5%)	-0.2	-14.7	11.1	0.780
Non-health benefits (n = 83)	68	(81.9%)	7.6*	-4.4	19.6	0.210
Personal agency (n = 97)	71	(73.2%)	-1.1	-13.3	11.0	0.856
All (n = 442)	333	(75.3%)				
**“Booster doses of COVID-19 vaccine are necessary to protect my health”**						
Control (n = 109)	86	(78.9%)	ref			
Personal health benefits (n = 73)	61	(83.6%)	4.7	-7.1	16.5	0.434
Community health benefits (n = 80)	60	(75.0%)	-3.9	-16.1	8.3	0.528
Non-health benefits (n = 83)	67	(80.7%)	1.8	-9.8	13.4	0.756
Personal agency (n = 97)	74	(76.3%)	-2.6	-14.1	8.9	0.653
All (n = 442)	348	(78.7%)				
**“Booster doses of COVID-19 vaccine are necessary to protect other people’s health”**						
Control (n = 109)	82	(75.2%)	ref			
Personal health benefits (n = 73)	60	(82.2%)	7.0*	-5.4	19.3	0.266
Community health benefits (n = 80)	60	(75.0%)	-0.2	-12.9	12.4	0.971
Non-health benefits (n = 83)	68	(81.9%)	6.7*	-5.2	18.6	0.266
Personal agency (n = 97)	72	(74.2%)	-1.0	-13.0	11.0	0.869
All (n = 442)	342	(77.4%)				
**“If I don’t get a booster dose of COVID-19 vaccine, I may get COVID-19”**						
Control (n = 109)	67	(61.5%)	ref			
Personal health benefits (n = 73)	47	(64.4%)	2.9	-11.6	17.4	0.690
Community health benefits (n = 80)	52	(65.0%)	3.5	-10.6	17.6	0.619
Non-health benefits (n = 83)	54	(65.1%)	3.6	-10.3	17.5	0.609
Personal agency (n = 97)	62	(63.9%)	2.5	-10.9	15.8	0.717
All (n = 442)	282	(63.8%)				

^**§**^ Percentage point difference

*Indicates a difference ≥5 percentage points between the intervention group and the control group

In the sub-analysis of hesitant participants, fewer hesitant participants agreed with beliefs compared to the whole study population. A slight majority of hesitant participants agreed that booster doses protect their health (55.7%, 93/167) and the health of others (53.9%, 90/167). In contrast, a slight minority of hesitant participants agreed that booster doses are safe (43.1%, 72/167), that booster doses prevent disease (48.5%, 81/167), or that they were at risk of getting COVID-19 without a booster dose (46.1%, 77/167). Looking at specific intervention groups, there were more differences ≥5 percentage points compared to the control in hesitant participants compared to the whole study population, although there is a lack of evidence in support of these findings. The full results are included in S1 File.

## Discussion

This experiment found some evidence that messages emphasising non-health benefits of getting a COVID-19 booster dose, like travelling, enjoying family occasions like weddings, and seeing family and friends, may increase intention to vaccinate, especially in hesitant populations. In this study, intention was qualitatively higher in participants who viewed messages about non-health benefits compared to those who viewed messages about health benefits for themselves and the community. This is somewhat consistent with findings from an experiment in US adults [25], which found some evidence of persuasive messages about non-health benefits being effective, however not more so than other persuasive strategies. This finding from the current study could be explained by the point in time in which this experiment was conducted. At the time, the Australian population had endured almost two years of public health restrictions, including lockdowns and international and domestic border closures. Being able to live more normally, free from restrictions, may have been top of mind, and hence what participants found the most persuasive. Equally, participants may have found the benefits of vaccination beyond the prevention of severe disease more appealing, especially younger participants. While evidence from a cross-national study early in the pandemic suggested age was not associated with risk perceptions relating to COVID-19 [42], Australian participants in this current study may have experienced or observed others experiencing mild COVID-19 during the first Omicron wave in Australia in December 2021 and thus may not have felt vulnerable to the severe effects of COVID-19.

This study also found some evidence that messages emphasising benefits of COVID-19 vaccine booster doses to people’s personal health and the health of the community may increase intention to vaccinate. These findings are consistent with a systematic review of COVID-19 vaccine interventions [22]. Despite qualitatively increasing intention, however, reminding participants about the personal or community health benefits of booster doses was only slightly more effective than informing participants about their eligibility to receive a booster dose (the control message). This is consistent with findings from an experimental study that found that information about where to get a vaccine was not rendered more effective when combined with information about the community or personal benefits of getting vaccinated [43]. In keeping with conclusions from the systematic review [22], reminders about the health benefits of COVID-19 vaccination for one’s self and others may have had less of an effect due to participants’ over-familiarity with this information after more than 12 months of communication from health authorities.

This study found that messages emphasising personal agency may have negative impacts, especially with hesitant individuals. This finding contrasts with previous findings, which suggest that individual liberty and a sense of personal agency when choosing vaccination may motivate people to vaccinate [18, 19]. The emphasis this message placed on the act of choosing a booster dose as ‘the right decision’ may help explain this result. Hesitant individuals may have reacted negatively (psychological reactance) [44] to suggestions about how they ought to behave, and what others approve or disapprove of regarding vaccination decisions. Research suggests infringement on freedom is associated with less positive attitudes towards vaccination [45] and, in cases where vaccination mandates are applied, this may lead to lower intentions to vaccinate [46], although findings in this area are mixed [47]. Equally, liberty as a moral value driving vaccination decision-making may vary by country and political views and may not resonate strongly with Australian audiences.

Finally, in terms of COVID-19 vaccine booster dose beliefs, this study found more increased changes in beliefs in hesitant participants compared to the whole study population. This suggests that hesitant participants have beliefs that are possibly less fixed and more amenable to change, a result that has been found in studies about childhood vaccination [48]. This would not necessarily hold for staunch vaccine refusers, but rather individuals who are more undecided or “fence sitters” [49].

These have practical implications for communications from health authorities and other stakeholders to encourage uptake of COVID-19 booster doses, especially communications directed at individuals consuming vaccination information online. Health authorities should consider using a range of messages; an emphasis on non-health benefits may be particularly useful, although personal and community health benefits may also be effective. Any message should be pre-tested with target audiences; message effectiveness will depend on current circumstances and sentiment. Interventions specifically designed for and targeting hesitant, undecided populations are likely to bear the most fruit. For this population, messages emphasising personal agency should be used with caution. Until more evidence emerges, caution should also be taken with messages that leverage prescriptive norms that tell people how they ought to act (i.e., what the ‘right’ vaccination behaviour is). In all instances, messages should be tailored for specific communities, taking into account differences in factors affecting motivation to vaccinate and health literacy. Caution should be used when extrapolating this evidence for use with messages directed at individuals hesitant about other vaccines. Research with the community during the initial rollout of COVID-19 vaccines indicates the vaccines are perceived to be considerably different from other vaccines give their use of novel technologies (such as mRNA) and rapid development. Some individuals who report being accepting of most vaccines have indicated hesitancy in relation to COVID-19 vaccines [29, 30].

Future research could use qualitative methodologies to explore message development and testing to better understand the negative effects observed in this study. Research to better understand the effect of messages that leverage prescriptive norms would be useful. Exploring the effects of messaging on different age groups, as well as messaging that caters for varying literacy levels, would provide evidence to support development of more nuanced and targeted communications. Likewise, developing and testing different message formats beyond written information, such as videos and infographics, and channels beyond a static webpage would also be useful. Research focusing specifically on at-risk groups and hesitant populations would ensure greater vaccine equity and would provide useful insights into intervention effectiveness in key target populations. At-risk groups could include the elderly, pregnant women and people, people from different cultural or Indigenous communities, and people with disabilities. Several years into the COVID-19 pandemic, it would also be useful to understand ‘vaccine information fatigue’ (i.e., the extent to which communities have switched off from and appear immune to communication about COVID-19 vaccines), and how to continue encouraging uptake. This is particularly important if health authorities are to consider additional booster doses or an annual COVID-19 vaccine. Furthermore, given the overall high intentions found among the sample in this study, future research may need to shift the focus to interventions that translate intention into vaccination behaviour (i.e., people getting vaccinated). This could involve the use of behavioural nudges, such as text message reminders, which has shown some positive results early in the COVID-19 vaccination rollout, as well as with other vaccines such as influenza [38, 43].

This study has limitations. In this study, participants only saw the message once from a single source; multiple exposures from multiple sources over time may be required for long term changes to intention and behaviour to occur. This study did not measure changes in behaviour, but rather measured vaccination intention as an outcome. While in keeping with similar studies, behaviour in the form of vaccination uptake would provide a more accurate measure of the effectiveness of such interventions. Some of the intervention texts had a slight overlap. There was a clear emphasis on a single area of focus in each text via a bolded title and repetition of the particular benefit throughout the text. Future research, however, should use a manipulation check to verify that participants can identify the condition they are in, as well as checks to assess comprehension. The sample was highly educated, likely a result of the nature of the panel used. Online research panels in general are likely to represent people who are digitally literate and are willing and able to spend time responding to email invitations and surveys [50]. While this group is the target population for this study, these results may not be generalisable to other populations, such as individuals with low health literacy or from culturally and linguistically diverse backgrounds. While the study is underpowered due to a post hoc variation to the protocol, the findings reported as difference in proportion is practically more meaningful and easier to communicate. Participants were quasi-randomised due to use of the sequence to prioritise the least filled group. Given equal distribution of participants among intervention groups, however, the threat to internal validity is limited. An appropriate, validated measure of hesitancy towards COVID-19 vaccines that did not include intention items was not available at the time of this study. As such, the measure used may not have accurately captured this construct. The study measured group differences rather than using a pre-post within-subject design, and thus did not capture changes in individual participants’ intentions after viewing persuasive messages. Checks to assess comprehension would further strengthen the study.

## Conclusions

Health authorities should consider emphasising the broader benefits of vaccination beyond prevention of severe disease to encourage uptake of COVID-19 vaccine booster doses, as well as benefits to people’s personal health and the health of the community. Communication using personal agency should be used with caution. The findings of this research can inform future communication about booster doses of COVID-19 vaccines.

## Supporting information

S1 Checklist CONSORT 2010 checklist of information to include when reporting a randomised trial*. (DOC)

S1 File Supporting information. (DOCX)

S2 File Protocol for persuasive COVID-19 vaccination message testing. (DOCX)

## References

[1] Moghadas SM, Vilches TN, Zhang K, Wells CR, Shoukat A, Singer BH, et al. The Impact of Vaccination on Coronavirus Disease 2019 (COVID-19) Outbreaks in the United States. Clin Infect Dis. 2021;73(12):2257–64. doi: 10.1093/cid/ciab079 33515252 PMC7929033

[2] Rossman H, Shilo S, Meir T, Gorfine M, Shalit U, Segal E. COVID-19 dynamics after a national immunization program in Israel. Nat Med. 2021;27(6):1055–61. doi: 10.1038/s41591-021-01337-2 33875890

[3] Lustig Y, Gonen T, Meltzer L, Gilboa M, Indenbaum V, Cohen C, et al. Superior immunogenicity and effectiveness of the third compared to the second BNT162b2 vaccine dose. Nat Immunol. 2022;23(6):940–6. doi: 10.1038/s41590-022-01212-3 35534723

[4] Australian Government Department of Health and Aged Care. Vaccination numbers and statistics [Internet]. 2022 [cited 2022 8 September]. Available from: https://www.health.gov.au/initiatives-and-programs/covid-19-vaccines/numbers-statistics

[5] Government of the United Kingdom. Vaccination in United Kingdom 2022 [cited 2022 8 September]. Available from: https://coronavirus.data.gov.uk/details/vaccinations

[6] European Centre for Disease Prevention and Control. COVID-19 vaccine tracker 2022 [cited 2022 8 September]. Available from: https://vaccinetracker.ecdc.europa.eu/public/extensions/COVID-19/vaccine-tracker.html#uptake-tab

[7] Centers for Disease Control (US Department of Health and Human Services). COVID data tracker 2022 [cited 2022 8 September]. Available from: https://covid.cdc.gov/covid-data-tracker/#vaccinations_vacc-people-additional-dose-totalpop

[8] Brewer NT, Chapman GB, Rothman AJ, Leask J, Kempe A. Increasing Vaccination: Putting Psychological Science Into Action. Psychological Science in the Public Interest. 2017;18(3):149–207. doi: 10.1177/1529100618760521 29611455

[9] Commonwealth of Australia. Review of Australia’s Health Sector Response to Pandemic (H1N1) 2009: Lessons Identified Canberra; 2011.

[10] Peretti-Watel P, Seror V, Cortaredona S, Launay O, Raude J, Verger P, et al. A future vaccination campaign against COVID-19 at risk of vaccine hesitancy and politicisation. The Lancet Infectious Diseases. 2020;20(7):769–70. doi: 10.1016/S1473-3099(20)30426-6 32445713 PMC7239623

[11] Bolsewicz KT, Steffens MS, Bullivant B, King C, Beard F. "To Protect Myself, My Friends, Family, Workmates and Patients…and to Play My Part": COVID-19 Vaccination Perceptions among Health and Aged Care Workers in New South Wales, Australia. Int J Environ Res Public Health. 2021;18(17):8954. 34501545 10.3390/ijerph18178954 PMC8431016

[12] Steffens MS, Bullivant B, Bolsewicz K, King C, Beard F. Factors Influencing COVID-19 Vaccine Acceptance in High Income Countries Prior to Vaccine Approval and Rollout: A Narrative Review. Int J Public Health. 2022;67:1604221. doi: 10.3389/ijph.2022.1604221 35250430 PMC8888412

[13] Freeman D, Loe BS, Yu LM, Freeman J, Chadwick A, Vaccari C, et al. Effects of different types of written vaccination information on COVID-19 vaccine hesitancy in the UK (OCEANS-III): a single-blind, parallel-group, randomised controlled trial. Lancet Public Health. 2021;6(6):e416–e27. doi: 10.1016/S2468-2667(21)00096-7 33991482 PMC8116130

[14] Betsch C, Bohm R, Korn L. Inviting free-riders or appealing to prosocial behavior? Game-theoretical reflections on communicating herd immunity in vaccine advocacy. Health Psychol. 2013;32(9):978–85. doi: 10.1037/a0031590 24001248

[15] Betsch C, Böhm R, Korn L, Holtmann C. On the benefits of explaining herd immunity in vaccine advocacy. Nature Human Behaviour. 2017;1(3).

[16] Bohm R, Betsch C. Prosocial vaccination. Curr Opin Psychol. 2022;43:307–11. doi: 10.1016/j.copsyc.2021.08.010 34517200

[17] Chapman G, Al Imam MH, Khan A, Smoll N, Adegbija O, Kirk M, et al. "Scary to get, more scary not to": COVID-19 vaccine acceptance among healthcare workers in Central Queensland, Australia, a cross-sectional survey. Commun Dis Intell (2018). 2022;46. doi: 10.33321/cdi.2022.46.30 35591752

[18] Schmidtke KA, Kudrna L, Noufaily A, Stallard N, Skrybant M, Russell S, et al. Evaluating the relationship between moral values and vaccine hesitancy in Great Britain during the COVID-19 pandemic: A cross-sectional survey. Soc Sci Med. 2022;308:115218. doi: 10.1016/j.socscimed.2022.115218 35870299 PMC9281411

[19] Amin AB, Bednarczyk RA, Ray CE, Melchiori KJ, Graham J, Huntsinger JR, et al. Association of moral values with vaccine hesitancy. Nat Hum Behav. 2017;1(12):873–80. doi: 10.1038/s41562-017-0256-5 31024188

[20] Jarrett C, Wilson R, O’Leary M, Eckersberger E, Larson HJ, Sage Working Group on Vaccine Hesitancy. Strategies for addressing vaccine hesitancy—A systematic review. Vaccine. 2015;33(34):4180–90. 25896377 10.1016/j.vaccine.2015.04.040

[21] World Health Organization. Data for action: achieving high uptake of COVID-19 vaccines. Geneva; 2021.

[22] Batteux E, Mills F, Jones LF, Symons C, Weston D. The Effectiveness of Interventions for Increasing COVID-19 Vaccine Uptake: A Systematic Review. Vaccines (Basel). 2022;10(3). doi: 10.3390/vaccines10030386 35335020 PMC8949230

[23] Ashworth M, Thunstrom L, Cherry TL, Newbold SC, Finnoff DC. Emphasize personal health benefits to boost COVID-19 vaccination rates. Proceedings of the National Academy of Sciences of the United States of America. 2021;118(32). doi: 10.1073/pnas.2108225118 34315824 PMC8364198

[24] Berliner Senderey A, Ohana R, Perchik S, Erev I, Balicer R. Encouraging Uptake of the COVID-19 Vaccine Through Behaviorally Informed Interventions: National Real-World Evidence From Israel. SSRN Electronic Journal. 2021.

[25] James EK, Bokemper SE, Gerber AS, Omer SB, Huber GA. Persuasive messaging to increase COVID-19 vaccine uptake intentions. Vaccine. 2021;39(49):7158–65. doi: 10.1016/j.vaccine.2021.10.039 34774363 PMC8531257

[26] Pfattheicher S, Petersen MB, Bohm R. Information about herd immunity through vaccination and empathy promote COVID-19 vaccination intentions. Health Psychol. 2022;41(2):85–93. doi: 10.1037/hea0001096 34570535

[27] Trueblood JS, Sussman AB, O’Leary D. The Role of Risk Preferences in Responses to Messaging About COVID-19 Vaccine Take-Up. Social Psychological and Personality Science. 2021;13(1):311–9.

[28] Baker MG, Wilson N, Blakely T. Elimination could be the optimal response strategy for covid-19 and other emerging pandemic diseases. BMJ. 2020;371:m4907. doi: 10.1136/bmj.m4907 33561814

[29] Bullivant B, Bolsewicz KT, King C, Steffens MS. COVID-19 vaccination acceptance among older adults: A qualitative study in New South Wales, Australia. Public Health Pract (Oxf). 2023;5:100349. doi: 10.1016/j.puhip.2022.100349 36532098 PMC9737511

[30] Steffens MS, Bullivant B, King C, Bolsewicz K. "I’m scared that if I have the vaccine, it’s going to make my lung condition worse, not better." COVID-19 vaccine acceptance in adults with underlying health conditions—A qualitative investigation. Vaccine X. 2022;12:100243. doi: 10.1016/j.jvacx.2022.100243 36447620 PMC9686055

[31] Kaufman J, Steffens MS, Hoq M, King C, Marques MD, Mao K, et al. Effect of persuasive messaging about COVID-19 vaccines for 5- to 11-year-old children on parent intention to vaccinate. J Paediatr Child Health. 2023;59(4):686–93. doi: 10.1111/jpc.16374 36807943

[32] Australian Bureau of Statistics. COVID-19 Mortality by wave. Australian Bureau of Statistics [Internet]. 16 Nov 2022 Date accessed 6 March 2023. Available from: https://www.abs.gov.au/articles/covid-19-mortality-wave.

[33] Sas N, Timms P. Omicron COVID-19 variant: Experts say vaccine protection against severe disease remains high, but spread will continue. Australian Broadcasting Corporation [Internet]. 15 Dec 2021 Date accessed 10 March 2023. Available from: https://www.abc.net.au/news/2021-12-15/omicron-variant-australia-covid-how-severe-vaccine-protection/100701012.

[34] Corben P, Leask J. Vaccination hesitancy in the antenatal period: a cross-sectional survey. BMC public health. 2018;18(1):566. doi: 10.1186/s12889-018-5389-6 29716556 PMC5930425

[35] McDonald C, Leask J, Chad N, Danchin M, Fethney J, Trevena L. A Consent Support Resource with Benefits and Harms of Vaccination Does Not Increase Hesitancy in Parents-An Acceptability Study. Vaccines (Basel). 2020;8(3). doi: 10.3390/vaccines8030500 32887503 PMC7565597

[36] Nyhan B, Reifler J. Does correcting myths about the flu vaccine work? An experimental evaluation of the effects of corrective information. Vaccine. 2015;33(3):459–64. doi: 10.1016/j.vaccine.2014.11.017 25499651

[37] Cohen J. Statistical Power Analysis for the Behavioral Sciences. New York: Routledge; 1988.

[38] Milkman KL, Patel MS, Gandhi L, Graci HN, Gromet DM, Ho H, et al. A megastudy of text-based nudges encouraging patients to get vaccinated at an upcoming doctor’s appointment. Proceedings of the National Academy of Sciences of the United States of America. 2021;118(20). doi: 10.1073/pnas.2101165118 33926993 PMC8157982

[39] Pink SL, Chu J, Druckman JN, Rand DG, Willer R. Elite party cues increase vaccination intentions among Republicans. Proceedings of the National Academy of Sciences of the United States of America. 2021;118(32). doi: 10.1073/pnas.2106559118 34312254 PMC8364165

[40] Tironi PA, Barham E, Zuckerman Daly S, Gerez JE, Marshall J, Pocasangre O. Messages that increase COVID-19 vaccine acceptance: Evidence from online experiments in six Latin American countries. PloS one. 2021;16(10):e0259059. doi: 10.1371/journal.pone.0259059 34710168 PMC8553119

[41] Amrhein V, Greenland S, McShane B. Scientists rise up against statistical significance. Nature. 2019;567(7748):305–7. doi: 10.1038/d41586-019-00857-9 30894741

[42] Dryhurst S, Schneider CR, Kerr J, Freeman AL, Recchia G, Van Der Bles AM, et al. Risk perceptions of COVID-19 around the world. Journal of Risk Research. 2020;23(7–8):994–1006.

[43] Dai H, Saccardo S, Han MA, Roh L, Raja N, Vangala S, et al. Behavioural nudges increase COVID-19 vaccinations. Nature. 2021;597(7876):404–9. doi: 10.1038/s41586-021-03843-2 34340242 PMC8443442

[44] Brehm SS, Brehm JW. Psychological Reactance: A Theory of Freedom and Control. Burlington: Elsevier Science; 2013.

[45] Hornsey MJ, Harris EA, Fielding KS. The psychological roots of anti-vaccination attitudes: A 24-nation investigation. Health Psychol. 2018;37(4):307–15. doi: 10.1037/hea0000586 29389158

[46] Sprengholz P, Betsch C, Bohm R. Reactance revisited: Consequences of mandatory and scarce vaccination in the case of COVID-19. Appl Psychol Health Well Being. 2021;13(4):986–95. doi: 10.1111/aphw.12285 34032388 PMC8239828

[47] Albarracin D, Jung H, Song W, Tan A, Fishman J. Rather than inducing psychological reactance, requiring vaccination strengthens intentions to vaccinate in US populations. Scientific Reports. 2021;11(1):1–9. 34675256 10.1038/s41598-021-00256-z PMC8531364

[48] Betsch C, Korn L, Holtmann C. Don’t try to convert the antivaccinators, instead target the fence-sitters. Proceedings of the National Academy of Sciences. 2015;112(49):E6725. doi: 10.1073/pnas.1516350112 26598650 PMC4679061

[49] Leask J. Target the fence-sitters. Nature. 2011;473(7348):443. doi: 10.1038/473443a 21614055

[50] Loosveldt G, Sonck N. An evaluation of the weighting procedures for an online access panel survey. Survey Research Methods. 2008;2(2).

