# Supplementary material for: Testing persuasive messages about booster doses of COVID-19 vaccines on intention to vaccinate in Australian adults: A randomised controlled trial
Source: PLoS One. 2023 Jun 2;18(6):e0286799. doi: 10.1371/journal.pone.0286799 (PMC10237633; doi:10.1371/journal.pone.0286799)
Supplement: S1 File — (DOCX) [file pone.0286799.s002.docx]

**Supporting information for: Steffens et al. Testing persuasive messages about booster doses of COVID-19 vaccines on intention to vaccinate in Australian adults: A randomised controlled trial**

Table S1. Original frequency distribution of intention to vaccinate by intervention group

|  | Intention (‘How likely is it that you will get a booster dose of COVID-19 vaccine?’) | | | | | | | | | | |
| --- | --- | --- | --- | --- | --- | --- | --- | --- | --- | --- | --- |
|  | Total | Definitely not | | Probably not | | I’m not sure | | Probably | | Definitely | |
| **Group** | n | n | % | n | % | n | % | n | % | n | % |
| Control | 109 | 1 | 0.9 | 6 | 5.5 | 12 | 11.0 | 18 | 16.5 | 72 | 66.1 |
| Personal health | 73 | 3 | 4.1 | 3 | 4.1 | 4 | 5.5 | 9 | 12.3 | 54 | 74.0 |
| Community health | 80 | 4 | 5.0 | 1 | 1.3 | 6 | 7.5 | 12 | 15.0 | 57 | 71.3 |
| Non-health benefits | 83 | 1 | 1.2 | 2 | 2.4 | 4 | 4.8 | 10 | 12.0 | 66 | 79.5 |
| Personal agency | 97 | 3 | 3.1 | 2 | 2.1 | 16 | 16.5 | 10 | 10.3 | 66 | 68.0 |
| All | 442 | 12 | 2.7 | 14 | 3.2 | 42 | 9.5 | 59 | 13.3 | 315 | 71.3 |

Table S2. Comparing beliefs between intervention groups and the control group in hesitant participants

| **Beliefs** | Strongly/slightly agree  n (%) | | Diff**^§^** | 95% lower | 95% upper | *p* |
| --- | --- | --- | --- | --- | --- | --- |
| **“Booster doses of COVID-19 vaccine are safe”** | | | | | | |
| Control (n=38) | 15 | (39.5%) | ref |  |  |  |
| Personal health (n=24) | 13 | (54.2%) | 14.7* | -11.4 | 40.8 | 0.257 |
| Community health (n=30) | 14 | (46.7%) | 7.2* | -17.2 | 31.6 | 0.552 |
| Non-health benefits (n=33) | 14 | (42.4%) | 3.0 | -20.7 | 26.6 | 0.801 |
| Personal agency (n=42) | 16 | (38.1%) | -1.4 | -23.4 | 20.6 | 0.899 |
| **“Booster doses of COVID-19 vaccine do a good job preventing disease”** | | | | | | |
| Control (n=38) | 16 | (42.1%) | ref |  |  |  |
| Personal health (n=24) | 11 | (45.8%) | 3.7 | -22.5 | 30.0 | 0.773 |
| Community health (n=30) | 14 | (46.7%) | 4.6 | -20.0 | 29.1 | 0.707 |
| Non-health benefits (n=33) | 20 | (60.6%) | 18.5* | -5.2 | 42.2 | 0.120 |
| Personal agency (n=42) | 20 | (47.6%) | 5.5* | -16.9 | 40.8 | 0.621 |
| **“Booster doses of COVID-19 vaccine are necessary to protect my health”** | | | | | | |
| Control (n=38) | 19 | (50.0%) | ref |  |  |  |
| Personal health (n=24) | 15 | (62.5%) | 12.5* | -13.7 | 38.7 | 0.335 |
| Community health (n=30) | 15 | (50.0%) | 0.0 | -24.7 | 24.7 | 1.000 |
| Non-health benefits (n=33) | 22 | (66.7%) | 16.7* | -6.8 | 40.1 | 0.156 |
| Personal agency (n=42) | 22 | (52.4%) | 2.4 | -20.2 | 24.9 | 0.832 |
| **“Booster doses of COVID-19 vaccine are necessary to protect other people’s health”** | | | | | | |
| Control (n=38) | 16 | (42.1%) | ref |  |  |  |
| Personal health (n=24) | 15 | (62.5%) | 20.4* | -5.6 | 46.4 | .118 |
| Community health (n=30) | 16 | (53.3%) | 11.2* | -13.3 | 35.8 | .357 |
| Non-health benefits (n=33) | 21 | (63.6%) | 21.5* | -2.0 | 45.0 | .070 |
| Personal agency (n=42) | 22 | (52.4%) | 10.3* | -12.1 | 32.7 | .358 |
| **“If I don’t get a booster dose of COVID-19 vaccine, I may get COVID-19”** | | | | | | |
| Control (n=38) | 15 | (39.5%) | ref |  |  |  |
| Personal health (n=24) | 10 | (41.7%) | 2.2 | -23.8 | 28.2 | .864 |
| Community health (n=30) | 13 | (43.3%) | 3.9 | -20.5 | 28.2 | .748 |
| Non-health benefits (n=33) | 19 | (57.6%) | 18.1* | -5.6 | 41.8 | .128 |
| Personal agency (n=42) | 20 | (47.6%) | 8.1* | -14.2 | 30.5 | .463 |

**^§^** Percentage point difference

*Indicates a difference ≥5% between the intervention group and the control group

Table S3. Comparing intention to vaccinate between intervention groups and the control group in non-hesitant participants (sub-analysis)

| Group | **Total n** | **Definitely/ probably intend on getting a booster dose** | **%** | **Diff^§^** | **95% CI Lower** | **95% CI Upper** | ***p*** |
| --- | --- | --- | --- | --- | --- | --- | --- |
| Control | 71 | 66 | 93.0 | - | - | - | - |
| Personal health benefits | 49 | 47 | 95.9 | 3.0 | -5.7 | 11.6 | 0.501 |
| Community health benefits | 50 | 49 | 98.0 | 5.0* | -2.9 | 13.0 | 0.212 |
| Non-health benefits | 50 | 50 | 100 | 7.0* | -0.2 | 14.3 | 0.056 |
| Personal agency | 55 | 54 | 98.2 | 5.2* | -2.4 | 12.8 | 0.175 |
| All participants | 275 | 266 | 96.7 |  |  |  |  |

^§^ Percentage point difference

*Indicates a difference ≥5 percentage points between the intervention group and the control group
