# Supplementary material for: Testing persuasive messages about booster doses of COVID-19 vaccines on intention to vaccinate in Australian adults: A randomised controlled trial
Source: PLoS One. 2023 Jun 2;18(6):e0286799. doi: 10.1371/journal.pone.0286799 (PMC10237633; doi:10.1371/journal.pone.0286799)
Supplement: S2 File — (DOCX) [file pone.0286799.s003.docx]

**Protocol for Persuasive COVID-19 Vaccination Message testing**

Abstract

**Objective**: The aim of this study is to test the impact of different messages about COVID-19 vaccines in adults vaccinated against COVID-19 on intention to receive a COVID-19 vaccine booster doses.

**Methods**: We will recruit adults vaccinated against COVID-19 for this study. Participants will receive 1 of 4 information conditions or a control. The 4 information conditions will focus on:

- personal health impacts of vaccinating
- family and community health impacts of vaccinating
- non-health benefits of vaccinating (freedom)
- personal choice and control (liberty)

The control will be text about COVID-19 vaccination disseminated by health authorities.

**Results**: Analysis will compare the primary outcome measure (intention to receive a COVID-19 vaccine or booster dose) between intervention groups and the control (4 comparisons) using an ANOVA test. Secondary analyses will compare secondary outcome measures (beliefs about COVID-19 vaccines) between intervention groups and the control using an ANOVA test.

1. Background

Uptake of COVID-19 vaccines continues to be critical for controlling the COVID-19 pandemic and returning society to normal functioning (WHO 2020; Yamey 2020). Acceptance of COVID-19 vaccines is essential to achieve high uptake; low acceptance can compromise uptake if not addressed. This has been the experience with other pandemic vaccines, for example, the 2009 H1N1 influenza pandemic vaccine (Commonwealth of Australia 2011; Peretti-Watel 2020).

Information interventions are one strategy to support acceptance of COVID-19 vaccines (Brewer 2018; Jarrett 2015; WHO 2021). However, evidence on COVID-19 vaccine information interventions remains limited. This gap in evidence is important to address to support uptake of COVID-19 vaccines. Therefore, **the** **aim of this study is to test the impact of different messages about COVID-19 vaccination on intention to receive a COVID-19 vaccine and beliefs about COVID-19 vaccines.**

*Expected outcomes*: We expect to establish new evidence about the impact of information interventions on COVID-19 vaccine intentions and vaccine beliefs. Findings from this study will fill a gap in knowledge about how different information interventions affect COVID-19 vaccine intentions and beliefs.

2. Methodology

We propose an experiment with random allocation using post-intervention testing to determine the effect of different types of messages on intention to vaccinate and COVID-19 vaccine beliefs compared to a control.

*Participants*

Participants will need to meet the following criteria:

- 18 years or older
- living in Australia
- have had at least one dose of a COVID-19 vaccine
- competent in reading and writing in English

Excluded:

- Individuals who are medically exempt from COVID-19 vaccination
- Have had a booster dose of COVID-19 vaccine

This study is part of a larger study investigating factors influencing COVID-19 vaccine acceptance, and messaging to support acceptance of COVID-19 vaccines. This study obtained ethics approval from the Sydney Children's Hospitals Network (SCHN) Human Research Ethics Committee (HREC) (reference number: 2021/ETH00181).

Recruitment plan

We will recruit participants via a commercial research company. We will use the company Quality Online Research (QOR), which has access to an online panel. QOR is a member of the Australian Market and Social Research Society, whose members are required to abide by a code of professional conduct. This code requires the company to seek informed consent; to inform participants that participation is entirely voluntary; that they may withdraw from the study at any time; that data collected is non-identifiable and is stored appropriately. Members of the research team have worked successfully with this company before.

We will invite eligible panel members to the study via email. To avoid self-selection bias, the initial invitation email will include only broad details about the study. Individuals can indicate their interest by clicking on an ‘I am interested in participating’ button embedded in the email. This will take them to an online page with the Participant Information Sheet and Consent Form, designed to ensure participants are adequately informed about the research and understand that participation is voluntary. Individuals who give written digital consent will be directed to the online experiment platform.

Participants will be rewarded points for participating in the study that can be converted into gift cards, cash or donated to charity. QOR awards panellists approximately $1 per 5 minutes of survey length. When the amount accrues to $20, this can be redeemed as an eGiftcard, as a PayPal cash transfer to a nominated account or as a cheque. Panel members can nominate to donate their amounts to a selection of charities. By completing surveys, panellists also earn entry into an annual cash prize draw ($5,000: 1 x $2,000, 1 x $1,000, 10 x $100, 10 x $500 – all can be redeemed by eGiftcard via Paypal, Cheque, donation to a nominated charity).

Relevant recruitment materials include:

- email letter of invite
- Participant Information Sheet
- Consent Form

Study setting

We will collect data via an online platform.

Study steps

1. After consenting to participate, participants will answer screening questions based on the inclusion and exclusion criteria. Individuals who do not meet the inclusion criteria, or meet the exclusion criteria, will be excluded.
2. Using a survey format, we will collect socio-demographic data from participants including age, gender, education, state of residence, as well as vaccination status, to enable us to characterise and describe our participants in subsequent reports.
3. We will then ask participants a stratifying question to enable us to divide participants into ‘accepting’ and ‘hesitant’ sub-groups for sub-analysis.
4. Participants will be randomly assigned to receive one of the test messages or a control text.
5. After the intervention, we will ask participants to answer survey items to measure pre-defined outcomes. We will include attention check survey items to allow us to measure data quality and exclude participants who provide low quality or unreliable data.

Sample size

We aim to recruit 480 vaccinated adults eligible for a booster dose of COVID-19 vaccine, allowing for a drop-out/poor quality response rate of approximately 10%. This will ensure a sample size of 430 participants. The experiment will assign participants to 1 of 4 intervention groups or a control (5 groups in total).

*Sample size justification:* Assuming 80% power and significance at 0.01, a study involving 430 participants (86 per intervention group) would be powered to detect a small difference in outcomes measures between groups (effect size d=0.2).

*Sample size calculations:* We performed sample size calculations using G*Power, a program for power analysis and sample size calculations. We used an ‘a Priori’ power analysis to calculate the sample size (n=430) required for the primary analysis, between group comparisons (the difference between two independent means). We specified 80% power, significance at 0.01, and effect size of d=0.2 (see Figure 1).

We have determined the significance (alpha) level at 0.01 to account for 4 planned contrasts (see analysis below).

We interpreted Cohen’s d using the following conventions:

- 0.2 represents a 'small' effect size
- 0.5 represents a 'medium' effect size
- 0.8 represents a 'large' effect size

**Figure 1: Screenshot of sample size calculations performed via G*Power**


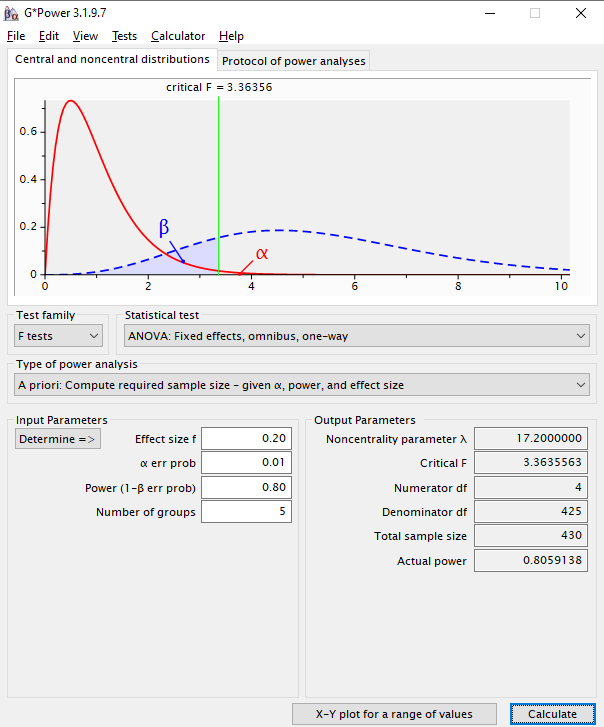


Data exclusion, data cleaning, missing data

**Data exclusion**: We will exclude data from any participant who:

- drops out
  - fails to complete the survey within 1 hour of starting
- provides poor quality data
  - completes the survey too quickly (to be determined by QOR)
  - fails to correctly answer attention check question
  - were determined to have provided low quality or unreliable data as determined by QOR quality checks.

**Data cleaning:** Other than those participants excluded for the reasons listed above, we do not intend to remove responses from participants for any other reason.

**Missing data**: We do not anticipate having missing data because participants must answer each survey question before they are able to move on to subsequent pages. As stated above, if a participant fails to complete the survey within 1 hour of starting, we will exclude their data from the analysis.

Intervention

The information intervention will be a short piece of text designed to be read in a short amount of time (<2 minutes). Each message will be informed by evidence-based barriers to or facilitators of vaccine acceptance, and/or evidence-based strategies to increase vaccine acceptance/uptake.

We will have 5 information conditions: 4 persuasive information conditions and a control.

- Message focusing on personal health impacts of vaccinating
- Message focusing on family and community health impacts of vaccinating
- Message focusing on non-health benefits of vaccinating (freedom)
- Message focusing on personal choice and control (liberty, autonomy)
- Control (baseline information condition)

The rationale for these information conditions is supported by evidence from previous research (Betsch 2013; Betsch 2015; Betsch 2017; Bish 2011; Bohm 2021; Bolsewicz 2021; Brewer 2007; Bruine de Bruin 2019; Freeman 2020; Freeman 2021).

Survey items

Survey items will measure study population characteristics age, gender, education, state of residence, and COVID-19 vaccination status. A stratifying item will measure self-reported COVID-19 vaccine hesitancy. The primary outcome measure will be intention to vaccinate against COVID-19. Secondary outcome measures will include COVID-19 vaccine beliefs about safety, effectiveness, ability to protect health of self and others, and risk perception about contracting COVID-19 if unvaccinated. The survey items will also include an attention check question.

| **Category** | **Survey item** |
| --- | --- |
| **Age** | *How old are you?*  [open-ended]*  *required, inclusion criteria |
| **Gender** | *What is your gender?*  (Female, Male, Not specified) |
| **Education status** | *What is the highest level of education or training you have completed?*  I did not attend school;  I am still at school;  Year 12 or below;  University degree;  Other non-school qualifications (certificates diplomas);  I prefer not to answer. |
| **State of residence** | *Where do you currently live?*  (NSW, VIC, Qld, TAS, WA, SA, NT, ACT) |
| **Vaccination status** | *Have you received a first dose of a COVID-19 vaccine?*  (yes/no)*  *required, inclusion criteria  *Have you received a second dose of a COVID-19 vaccine?*  (yes/no/prefer not to answer)  *Have you received a booster dose (third dose) of a COVID-19 vaccine?*  (yes/no)*  *required, inclusion criteria |
| **Hesitancy** | How much do you agree with the following statement:  *“I feel hesitant about COVID-19 vaccines”*  (Strongly agree, slightly agree, neither agree nor disagree, slightly disagree, strongly disagree) |
| **Intention**  ***(Primary outcome measure)*** | *How likely is it that you will get a booster dose of COVID-19 vaccine?*  (Definitely, probably, I’m not sure, probably not, definitely not) |
| **Beliefs**  *(Secondary*  *outcome measure)* | How much do you agree with the following statements:  -Booster doses of COVID-19 vaccine are safe.  -Booster doses of COVID-19 vaccine do a good job preventing disease caused by COVID-19.  -Booster doses of COVID-19 vaccine are necessary to protect my health.  -Booster doses of COVID-19 vaccine are necessary to protect other people’s health.  -If I don’t get a booster dose of COVID-19 vaccine, I may get COVID-19.  (Strongly agree, slightly agree, neither agree nor disagree, slightly disagree, strongly disagree) |
| **Attention check** | Please select strongly disagree for this item.  (Strongly agree, slightly agree, neither agree nor disagree, slightly disagree, strongly disagree) |

Transformations

- For the stratifying question ***Hesitancy***, we will recode responses where (strongly agree, slightly agree, neither agree nor disagree) = hesitant, and (slightly disagree, strongly disagree) = Not hesitant
- For analysis of the primary outcome measure ***Intention***, we will recode responses where Definitely = 5, probably = 4, I’m not sure = 3, probably not =2, definitely not = 1
- For analysis of the secondary outcome measures ***Beliefs***, we will recode responses where Strongly Disagree = 1, Disagree = 2, Not sure = 3, Agree = 4, Strongly Agree = 5

***Data analysis***

*Primary analysis*: We will use an ANOVA to compare primary outcome measure ***Intention*** between the 4 intervention groups and the control (4 comparisons).

*Secondary analyses:* We will use an ANOVA to compare ***Beliefs*** between groups and the control (4 comparisons per belief).

*Sub-analysis:* In hesitant participants only (identified by the stratifying question), we will use an ANOVA to compare outcome measures (Intention, Beliefs) between groups and the control (4 comparisons per outcome measure).

References

Betsch C, Böhm R, Korn L. Inviting free-riders or appealing to prosocial behavior? Game-theoretical reflections on communicating herd immunity in vaccine advocacy. Health Psychol. 2013;32(9):978–85. doi:10.1037/a0031590.

Betsch C, Böhm R, Chapman GB. Using behavioral insights to increase vaccination policy effectiveness. Policy Insights Behav Brain Sci. 2015;2(1):61–73. doi:10.1177/2372732215600716.

Betsch C, Böhm R, Korn L, Holtmann C. On the benefits of explaining herd immunity in vaccine advocacy. Nat Hum Behav. 2017; https://doi.org/10.1038/s41562-017-0056

Bish A, Yardley L, Nicoll A, Michie S. Factors associated with uptake of vaccination against pandemic influenza: a systematic review. Vaccine. 2011;29(38):6472–84. doi:10.1016/j.vaccine.2011.06.107.

Bohm, Betsch 2021. Prosocial vaccination. Current Opinion in Psychology. https://www.sciencedirect.com/science/article/pii/S2352250X21001433

Bolsewicz KT, Steffens MS, Bullivant B, King C, Beard F. “To Protect Myself, My Friends, Family, Workmates and Patients …and to Play My Part”: COVID-19 Vaccination Perceptions among Health and Aged Care Workers in New South Wales, Australia. Int. J. Environ. Res. Public Health. 2021, 18(17), 8954; doi: 10.3390/ijerph18178954

Brewer, N. T., Chapman, G. B., Rothman, A. J., Leask, J. & Kempe, A. Increasing vaccination: putting psychological science into action. Psychol. Sci. Public Interest. 2018; 18, 149–207 .

Brewer NT, Chapman GB, Gibbons FX, Gerrard M, McCaul KD, Weinstein ND. Meta-analysis of the relationship between risk perception and health behavior: the example of vaccination. Health Psychol. 2007;26(2):136–45. doi:10.1037/0278-6133.26.2.136.

Bruine de Bruin W, Parker AM, Galesic M, Vardavas R. Reports of social circles’ and own vaccination behavior: a national longitudinal survey. Health Psychol. 2019;38(11):975-983. doi:10.1037/hea0000771.

Commonwealth of Australia. Review of Australia's Health Sector Response to Pandemic (H1N1) 2009: Lessions Identified [Internet]. Canberra;2011 [Available from: https://www1.health.gov.au/internet/publications/publishing.nsf/Content/review-2011-l/$File/lessons%20identified-oct11.pdf.

Freeman et al. Effects of different types of written vaccination information on COVID-19 vaccine hesitancy in the UK (OCEANS-III): a single-blind, parallel-group, randomised controlled trial. Lancet Infectious Diseases. 2021. 6(6), E416-E427. <https://doi.org/10.1016/S2468-2667(21)00096-7>

Jarrett, C., Wilson, R., O’Leary, M., Eckersberger, E. & Larson, H. J. Strategies for addressing vaccine hesitancy – a systematic review. Vaccine 33, 4180–4190 (2015).Johns Hopkins COVID behaviours dashboard (Data 1-15 Sept 2021) <https://covidbehaviors.org/>

Peretti-Watel P, Seror V, Cortaredona S, Launay O, Raude J, Verger P, et al. A future vaccination campaign against COVID-19 at risk of vaccine hesitancy and politicisation. The Lancet Infectious Diseases. 2020;20(7):769-70.

WHO. Public statement for collaboration on COVID-19 vaccine development [Internet]. 2020 [cited 17 March 2021]. Available from: https://www.who.int/news-room/detail/13-04-2020-public-statement-for-collaboration-on-covid-19-vaccine-development.

WHO. Data for action: achieving high uptake of COVID-19 vaccines. 2021; Geneva: World Health Organization. Retrieved from: <https://www.who.int/publications/i/item/WHO-2019-nCoV-vaccination-demand-planning-2021.1>

Yamey G, Schäferhoff M, Hatchett R, Pate M, Zhao F, McDade KK. Ensuring global access to COVID-19 vaccines. The Lancet. 2020;395(10234):1405-6.
