# Supplementary material for: Testing persuasive messages about booster doses of COVID-19 vaccines on intention to vaccinate in Australian adults: A randomised controlled trial
Source: PLoS One. 2023 Jun 2;18(6):e0286799. doi: 10.1371/journal.pone.0286799 (PMC10237633; doi:10.1371/journal.pone.0286799)
Supplement: Attachment — Submitted filename: Steffensetal_Response to reviewers_round2.docx [file pone.0286799.s005.docx]

**Response to reviewers (Peer review round 2, May 2023) for** ‘Testing persuasive messages about booster doses of COVID-19 vaccines on intention to vaccinate in Australian adults: A randomised controlled trial’

**Reviewer #2**

2.1 *Please clarify whether the test of two proportion (sample size and Line 275) is using Chi-square test or a logistic regression. If logistic regression, add details of covariates.*

**Our response**: In this study we used the z-test for two proportions since the sample sizes were large enough (> 30 in each group) and assumed that the data follows a normal distribution. We have updated the manuscript to reflect this.

The text in the Methods now reads:

*“The difference in proportion of participants intending to receive a COVID-19 vaccine and agreeing with COVID-19 vaccine booster dose belief statements was compared between the control group and each of the four intervention groups using tests of two proportions (z test).”*

2.2 *Line 254, sample size is based on a test (Chi-square test?), not 95% CI.*

**Our response**: We have updated the manuscript to remove reference to 95% CI and indicate that we used z tests.

The text in the Methods now reads:

*“In line with the post hoc variation to analyse participant’s intention to vaccinate and belief items as dichotomous variables, the sample size of 85 participants per intervention group was adequate to estimate a difference in proportion of 15 percentage points or more between two groups using two-sample test of proportion (z-test) with a level of significance of 0.05 and power of 80%.”*

2.3 *Figure 1 the format is not correct. Check the examples on PLOSONE.*

**Our response**: We have updated Figure 1 to be in keeping with Schulz KF, Altman DG, Moher D, for the CONSORT Group (2010). CONSORT 2010 Statement: Updated Guidelines for Reporting Parallel Group Randomised Trials. PLoS Med 7(3): e1000251. We have also updated in-text citations of the figures to be in keeping with PLoS ONE requirements.

**Reviewer #4**

*4.1 Even though I understand that dichotomizing the outcome variable makes interpretation easier, the authors should provide more information about it. The cut-off point is rather arbitrary – especially for the middle category. To get a better understanding about the effects of the treatments, it would be helpful to see the differences between the treatment groups for the distribution of the original variable as well. It would also help to understand how the treatment affects the intention of the respondents (e.g., the “definitely not” group might not differ at all between groups, but the “I’m not sure” might shift towards “probably”). This is highly relevant since “definitely not” and “I’m not sure” are very different answers which are treated the same by dichotomizing the variable. The authors should at least provide information about the original distribution in the Supplementary Materials.*

**Our response**: We have provided information about the original distribution of the intention variable by intervention group in the supporting information.

The text in the Methods now reads:

*“In response to the skewed distribution of responses, a post hoc variation to the pre-registration was made to analyse intention to vaccinate and participant responses to belief items as dichotomous variables. See supporting information for original frequency distribution of intention to vaccinate.”*

See the supporting information for the new table showing the original frequency distribution of intention to vaccinate.

**Journal Requirements**

*1.1 Please review your reference list to ensure that it is complete and correct. If you have cited papers that have been retracted, please include the rationale for doing so in the manuscript text, or remove these references and replace them with relevant current references. Any changes to the reference list should be mentioned in the rebuttal letter that accompanies your revised manuscript. If you need to cite a retracted article, indicate the article’s retracted status in the References list and also include a citation and full reference for the retraction notice.*

**Our response**: We have reviewed our reference list and can confirm that it is complete and correct.
